# Comparison of Sub-Ppm Instrument Response Suggests Higher Detection Limits Could Be Used to Quantify Methane Emissions from Oil and Gas Infrastructure

**DOI:** 10.3390/s24113407

**Published:** 2024-05-25

**Authors:** Stuart N. Riddick, Mercy Mbua, Ryan Brouwer, Ethan W. Emerson, Abhinav Anand, Elijah Kiplimo, Seunfunmi Ojomu, Jui-Hsiang Lo, Daniel J. Zimmerle

**Affiliations:** Methane Emission Technology Evaluation Center (METEC), The Energy Institute, Colorado State University, Fort Collins, CO 80523, USA; mercy.mbua@colostate.edu (M.M.); ryan.brouwer@colostate.edu (R.B.); ethan.emerson@colostate.edu (E.W.E.); a.anand@colostate.edu (A.A.); elijah.kiplimo@colostate.edu (E.K.); seunfunmi.ojomu@colostate.edu (S.O.); jui-hsiang.lo@colostate.edu (J.-H.L.); dan.zimmerle@colostate.edu (D.J.Z.)

**Keywords:** methane, instrumentation, detection limits, oil and gas, controlled release

## Abstract

Quantifying and controlling fugitive methane emissions from oil and gas facilities remains essential for addressing climate goals, but the costs associated with monitoring millions of production sites remain prohibitively expensive. Current thinking, supported by measurement and simple dispersion modelling, assumes single-digit parts-per-million instrumentation is required. To investigate instrument response, the inlets of three trace-methane (sub-ppm) analyzers were collocated on a facility designed to release gas of known composition at known flow rates between 0.4 and 5.2 kg CH_4_ h^−1^ from simulated oil and gas infrastructure. Methane mixing ratios were measured by each instrument at 1 Hertz resolution over nine hours. While mixing ratios reported by a cavity ring-down spectrometer (CRDS)-based instrument were on average 10.0 ppm (range 1.8 to 83 ppm), a mid-infrared laser absorption spectroscopy (MIRA)-based instrument reported short-lived mixing ratios far larger than expected (range 1.8 to 779 ppm) with a similar nine-hour average to the CRDS (10.1 ppm). We suggest the peaks detected by the MIRA are likely caused by a micrometeorological phenomenon, where vortex shedding has resulted in heterogeneous methane plumes which only the MIRA can observe. Further analysis suggests an instrument like the MIRA (an optical-cavity-based instrument with cavity size ≤10 cm^3^ measuring at ≥2 Hz with air flow rates in the order of ≤0.3 slpm at distances of ≤20 m from the source) but with a higher detection limit (25 ppm) could detect enough of the high-concentration events to generate representative 20 min-average methane mixing ratios. Even though development of a lower-cost, high-precision, high-accuracy instrument with a 25 ppm detection threshold remains a significant problem, this has implications for the use of instrumentation with higher detection thresholds, resulting in the reduction in cost to measure methane emissions and providing a mechanism for the widespread deployment of effective leak detection and repair programs for all oil and gas infrastructure.

## 1. Introduction

Methane gas is a relatively powerful greenhouse gas, with a global warming potential 25 times larger than carbon dioxide, and has been identified as a key gas for emissions reduction if countries are to meet the climate goals set out by the IPCC and the Global Methane Pledge [1,2,3]. Quantification of methane emissions is crucial to understanding the relative size of sources and the efficacy of mitigation strategies. Methane emission cannot be directly measured, so instead the rate of methane emission for point sources (mass per unit time) or flux for area sources (mass per unit area per unit time) must be calculated from a measure of methane abundance (mass per unit volume) in the air combined with some measure of the airflow past the methane sensor (volume of air per unit time) [4,5].

Enclosed chamber methods are routinely used to measure emissions from either small point sources or small area sources on the ground [6,7,8]. Either the rate of change of methane concentration within a sealed volume (static chamber) or the steady state concentration within a chamber with a flow of air passing through (dynamic chamber or Hi-Flow sampler) can be used to infer the emission rate. All chamber approaches require the source to be contained within the chamber and these are limited to emission sources that are physically small.

For ground-based measurements, downwind methods have been used to quantify methane emissions and approaches generally comprise methane concentration measurements coupled with meteorological and micrometeorological data to infer an emission or flux rate [9,10,11]. While standard methods are typically used to measure the meteorology and micrometeorology (i.e., a sonic anemometer), there is a degree of variability in instrumentation used to measure the methane abundance in the air. Metal oxide sensors [12,13,14] are inexpensive and have shown that they can measure near-background methane concentrations, but their reliability in changing environmental conditions and their slow response to changing methane concentrations makes them a relatively unreliable methane sensor type [15]. Tunable diode laser absorption spectrometer (TDLAS) systems use the absolute absorption of a laser that is tuned to the absorption wavelength of methane, to quantify the abundance of methane in air within a fixed volume cavity [16]. While TDLAS systems are considered more accurate than metal oxide sensors, they are relatively sensitive to changes in temperature which affect the size of the cavity and result in relatively high drift over diurnal cycles [17].

While other quantification methods exist (gas chromatography, LiDAR, infrared spectrometry), the currently accepted gold-standard approach to atmospheric methane concentration measurement is using a laser-based optical cavity (similar to TDLAS) by which the methane concentration is inferred from the rate of change of laser intensity as it is reflected many times inside the cavity. Several vendors market multi-pass laser-based methane instruments; however, technological details on individual instrument design and construction remain proprietorial, and therefore, it is currently unclear how vendors’ systems compare.

Methane analyzers with high precision and low detection limits are currently thought to be the most useful for quantifying emissions from a variety of sources, such as oil and gas infrastructure, landfills, and livestock. Typically, the most useful analyzers report dry-mole fractions of methane between 0 and 100 ppm with a precision of ~1 ppb (1σ over 10 s) [10,18,19,20,21,22]. As such, measurements considered reliable can only be conducted using expensive instrumentation ($30 k–$50 k). Due to the expense of these instruments, few publications have reported the response of co-located instruments to changes in atmospheric methane [18,19].

While fast-response methane analyzers are reliable, their cost limits the much-needed wide coverage of methane measurements such as in continuous monitoring, where multiple sensors at a production/midstream site or basin are required. This study aims to investigate whether different analyzers (with different flow rates, response time, accuracy, etc.) can achieve the same accuracy in detected concentrations and quantification. To investigate the response of different vendors’ instruments to changing methane concentrations, we conducted an inter-comparison of three multi-pass laser-based methane instruments. The aims of the inter-comparison are to 1. observe the response of each instrument to changing methane concentrations downwind of a controlled natural gas emission; 2. investigate whether high-precision instruments with ppb-level detection limits are necessary for mixing ratio quantification; and 3. test the impact of using instruments with higher detection limits on emissions quantification. To our knowledge this is the first time that testing current assumptions on methane instrument detection limits has been investigated.

## 2. Materials and Methods

### 2.1. Controlled Release Experiments

Colorado State University’s Methane Emission Technology Evaluation Center (METEC; 40.595° N, 105.139° W) is a facility that can simulate point and area source emissions at rates of between 1 and 100,000 g h^−1^ from over 300 points across the 4000-square-meter site. A mid-infrared laser absorption spectroscopy (MIRA) instrument and an off-axis integrated cavity output spectroscopy (OA-ICOS) instrument were deployed inside a 1 m^3^ climate-controlled (20 °C) chamber 20 m and 350° North from an emission point on a well head, 1 m above ground level (AGL), on METEC’s pad one and five on the 13 July 2023. Inlets for the MIRA and OA-ICOS were collocated at 1 m AGL and 1.5 m of tubing ran from the inlet to the instrument. The inlet to a cavity ring-down spectroscopy (CRDS) analyzer was also collocated with the MIRA-OA-ICOS inlets, but the instrument and inlet were connected by 100 m of 1/4” OD PTFE tubing as the CRDS required more protection from the weather than either the OA-ICOS or MIRA. These three instruments were primarily chosen as they were available for use and they are all commonly used for methane measurements, although they operate differently. The sample rate of each instrument was calculated from the instrument cell turnover rate and analyses were conducted on the average concentration during each period of cell turnover.

#### 2.1.1. Cavity Ring-Down Spectroscopy (CRDS)

The cavity ring-down spectroscopy (CRDS) instrument measures methane concentration as a function of the ‘ring-down’ time of the optical cavity inside the instrument. A laser pulse of fixed wavelength is directed into a highly reflective cavity filled with the methane air mixture which is to be measured. As the laser is reflected within the cavity, its intensity decreases, and the methane concentration in the air is determined from the rate of change of intensity over time, i.e., the ring-down time. CRDS instruments are typically more precise than other optical instruments, but with precision comes a heavier instrument, higher cost, and a relatively low time response.

The CRDS instrument used here has a dynamic range of 1.5 to 30 ppm CH_4_ with a precision of <0.1 ppb CH_4_ (1σ over 5 min average). The instrument is classified as bench top or rack mountable, meaning it is the least portable of the three instruments. It has a power consumption of 160 W, sample flow rate of ~40 sccm at 101 kPa, and optical cavity volume of ~200 cm^3^. At these flow rates, we estimate the cell turnover rate at 33 s. This instrument is unsuitable for field deployment and was kept in a climate-controlled office during the measurements. At the time of the measurement there was no calibration gas available in the dynamic measurement range of the CRDS.

#### 2.1.2. Off-Axis Integrated Cavity Output Spectroscopy (OA-ICOS)

The off-axis integrated cavity output spectroscopy (OA-ICOS) instruments were developed from CRDS methods to overcome the issues of weight, cost, and response time [20,21]. These instruments comprise a cavity with highly reflective mirrors but, unlike CRDS, the frequency of a laser is swept over the absorption frequency of methane and the corresponding absorption measured. The operation of the OA-ICOS avoids the time cost of the CRDS ‘ring-down’ time.

The OA-ICOS reports methane mixing ratio measurements between 0 and 100 ppm and precision of 4 ppb (1σ over 1 s). The OA-ICOS has a stated maximum response rate of 10 Hz, but tests show that this rate varies between 4 and 8 Hz. An internal air pump (rate reported in the manual 11 L min^−1^; measured rate 2.1 L min^−1^) is incorporated into the 0.12 m^3^ case and the unit’s total weight and power draw are 6 kg and 35 W, respectively, making the OA-ICOS very portable. Using the measured flow rate, we estimate the OA-ICOS cell turnover rate at 1.5 s. The optical cavity has an estimated volume of 50 cm^3^. Two-minute bump tests show that the OA-ICOS reported methane mixing ratios of 50.0 ppm (s.d. 0.4), 91.0 ppm (s.d. 0.1), and 907.9 ppm (s.d. 0.7) in response to calibration gases with methane mixing ratios of 50 ppm, 100 ppm, and 1000 ppm, respectively. All calibration standards were accurate to ±5%.

#### 2.1.3. Mid-Infrared Laser Absorption Spectroscopy (MIRA) Instrument

The mid-infrared laser absorption spectroscopy (MIRA) instrument comprises a mid-infrared (3.1–4.1 µm) solid-state laser reflecting multiple times (path length of 13 m) within an optical cell, resulting in a small, portable instrument. The MIRA is designed to be carried on a drone and measures between 0.02 and 10,000 ppm at a precision of <1 ppb (1σ over 1 s). The internal pump draws air (rate reported in the manual 3 to 5 L min^−1^; measured rate 0.3 L min^−1^) into an optical cavity with volume 10 cm^3^. Using the measured flow rate, we estimate the MIRA cell turnover rate at 2 s. The MIRA was the most portable instrument, with a volume of 0.003 m^3^, weight of 1.6 kg, and a power consumption of 1.5 W.

Two-minute bump tests show that the MIRA reported methane mixing ratios of 53.7 ppm (s.d. 0.9), 105.6 ppm (s.d. 0.2), and 1084 ppm (s.d. 1.2) in response to calibration gases with methane mixing ratios of 50 ppm, 100 ppm, and 1000 ppm, respectively. All calibration standards were accurate to ±5%.

#### 2.1.4. Experimental Method

Between the 10th and 13th of July 2023, controlled emissions of between 0.4 and 5.3 kg CH_4_ h^−1^ were released 1 m AGL from points 80 m (Pad 1) and 20 m (Pad 5) away from the instruments’ inlet (Table 1). Methane mixing ratio data were measured by the three instruments and reported at 1 Hertz throughout the day. Meteorological and micrometeorological data were collected by an R.M. Young (Traverse City, MI) sonic anemometer at 2 m AGL and 1 m away from the gas analyzers’ inlets.

### 2.2. Further Analysis

To identify differences/bias between data reported by the three analyzers, scatter-plots of the 20 min reported averages have been generated and metric of slope, intercept, *R*^2^, and bias calculated. The instruments have been anonymized to preserve impartiality. The bias (%) between two instruments is calculated (Equation (1)) from the slope of the regression forced through zero (*m*) [22].

(1) $$bias=\left(m-1\right)\times 100$$

To fulfil the second and third aims of this study (2. Investigate whether high-precision instruments with ppb-level detection limits are necessary for mixing ratio quantification; and 3. Test the impact of using instruments with higher detection limits on emissions quantification), further analysis was conducted on the methane mixing ratio measurement data. This includes modifying the MIRA measurement data to simulate instruments with a higher detection limit, then using these data to generate emissions estimates; full details are provided in the subsequent sections. The MIRA was the only instrument chosen for the further analysis as it was the only instrument that reported the very large (and unexpected) mixing ratios.

#### 2.2.1. Simulate the Impact of Instruments with a Higher Detection Threshold

To simulate methane instruments with higher detection thresholds, high-pass filters were used on the 2 Hz MIRA mixing ratio data with limits of 10, 25, 50, 100, 200, and 300 ppm, where any methane mixing ratio value below the threshold was replaced with a background concentration value of 2 ppm. Following typical methods for emission quantification [5,23,24], 20 min averaged methane mixing ratios were calculated. To investigate how similar the 20 min averaged filtered data are to the 20 min averaged unfiltered data, we calculated the slope (*m*) and coefficient of determination (*R*^2^) of the linear regression of each of the filtered datasets.

#### 2.2.2. Impact of Higher Detection Threshold on Emission Quantification

To test how a higher detection threshold could propagate through to emissions quantification, 20 min averaged data using unfiltered data were replaced by the 20 min averaged mixing ratio data using filtered data for the thresholds of 10, 25, 50, 100, 200, and 300 ppm during releases from July 10th, 2023 and July 13th, 2023. Emissions were calculated using the same meteorological data using the Gaussian plume equation (Equation (2)). Input data used to calculate the emission (*Q*, g s^−1^) are the 20 min averages of the wind speed (*u*, m s^−1^); wind direction (*WD*, °); air temperature (*T*, °C); methane concentration downwind of the source (*Χ*, µg m^−3^); location and height of the methane detector; background methane concentration (*Χ_b_*, µg m^−3^); and the Pasquill–Gifford stability class [4]. The Pasquill–Gifford stability class was calculated using the wind speed and solar irradiance [25], while the wind direction and relative locations (latitude, longitude) of the emission (height *h_s_*, m) and measurement points (height *z*, m) were used to calculate the lateral distance from the center of the plume (*y*, m), and the standard deviation of the lateral (*σ_y_*, m) and vertical (σ_z_, m) mixing ratio distributions [25].

(2) $$X=\frac{Q}{2\pi u\sigma_{y}\sigma_{z}}e^{-\frac{y^{2}}{{\left(2\sigma_{y}\right)}^{2}}}\left(e^{-\frac{{\left(z-h_{s}\right)}^{2}}{{\left(2\sigma_{z}\right)}^{2}}}+e^{-\frac{{\left(z+h_{s}\right)}^{2}}{{\left(2\sigma_{z}\right)}^{2}}}\right)$$

Emissions were only calculated when the instrument was downwind of the emission point (±10° wind direction). The percentage difference in the emission quantification was then calculated as the percentage difference between the calculated and known emission rates. The average, the 5th percentile, and the 95th percentile of the emissions were then calculated from the emissions of each set of filtered data.

## 3. Results

### 3.1. Observed Methane Mixing Ratios

**Figure 1:**
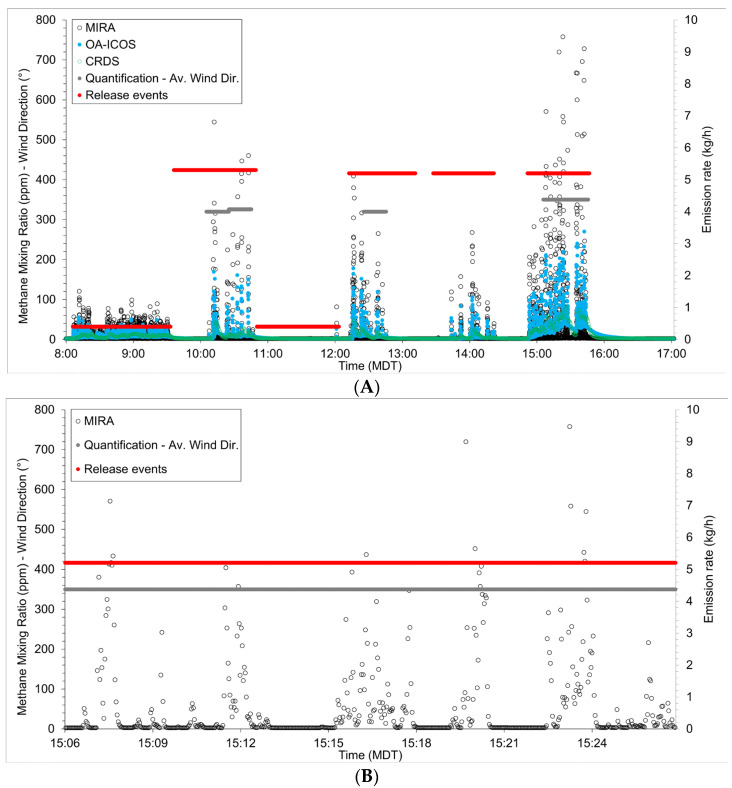
(**A**). Time series of the methane mixing ratios reported by all instruments during emission events 0800 to 1700 MT 13th July 2023. (**B**). Time series of the methane mixing ratios reported by the MIRA instruments during the emission event 1506 to 1526 MT 13th July 2023. Red line indicates the emission event and the grey line indicates the wind direction during the period of emission quantification.

During the release events, methane was detected by all three instruments when they were downwind of the points of release. Cell turnover rates (CRDS—33 s; OA-ICOS—2 s; MIRA—2 s) based on measured pump flow rates were used to filter data form independent mixing ratio values. Using these filtered data, the average mixing ratio over the nine hours detected by the CRDS was 9.97 ppm (maximum 83.00; minimum 2.16), by the OA-ICOS 10.00 ppm (maximum 301.62; minimum 2.16), and by the MIRA 10.11 ppm (maximum 778.90; minimum 1.94). The largest mixing ratios for all three instruments were detected during the largest emission event at the closest emission point (time—15:23 MDT; emission rate—5.2 kg h^−1^; Figure 1).

#### Comparison of Instrument-Reported Mixing Ratios

**Figure 2:**
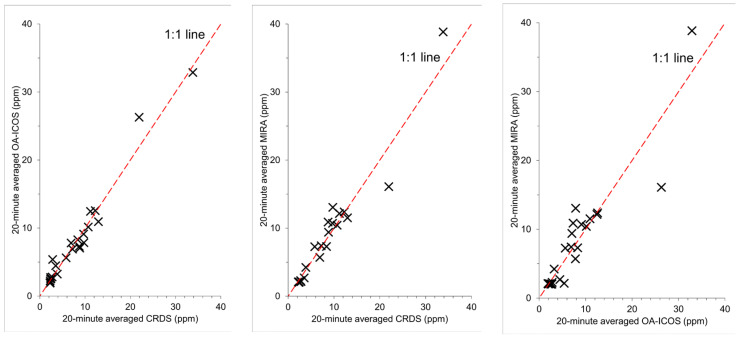
Scatter-plots of the 20 min averages of mixing ratios reported by each instrument.

Scatter-plots of the 20 min averaged methane mixing ratios from each of the instruments were plotted (Figure 2). Metrics generated from these plots (Table 2) show that the slope varies by ±0.05, the intercept from +0.44 ppm to −0.36 ppm, the *R*^2^ from 0.97 to 0.93, and the bias from 2.94% to −3.07%. Of the three instruments, the CRDS and OA-ICOS are in the best agreement (slope = 1.01, intercept = −0.09, *R*^2^ = 0.97, Bias = 0.23%) while comparisons of both the CRDS and OA-ICOS to the MIRA show similar results (Table 2).

### 3.2. Impact of Simulating Instruments with a Higher Detection Threshold

**Figure 3:**
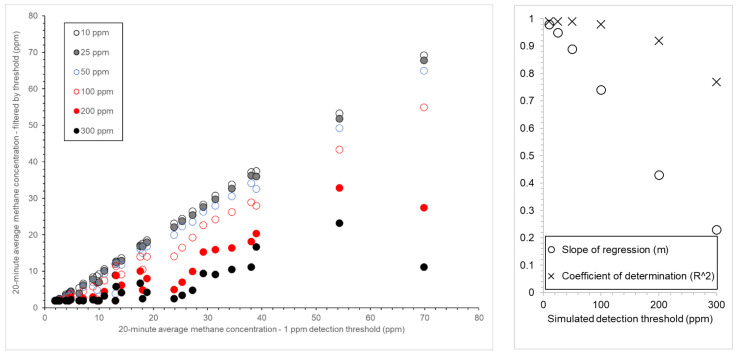
**Left pane**: the unfiltered 20 min average methane mixing ratios plotted against the filtered 20 min average methane mixing ratios, where a high-pass filter was used on the 2 Hz MIRA mixing ratio data to simulate instruments with detection thresholds of 10, 25, 50, 100, 200, and 300 ppm. **Right pane**: The slope (m) and coefficient of determination (*R*^2^) of the linear regression of each of the filtered datasets and the unfiltered dataset.

To investigate the effect of a high detection threshold, high-pass filters were used on the 2 Hz MIRA mixing ratio data with limits of 10, 25, 50, 100, 200, and 300 ppm and the 20 min average methane mixing ratios calculated. Linear regression between the unfiltered and filter 20 min averaged data show that as the simulated detection threshold increases, both the slope of the regression (*m*) and the coefficient of regression (*R*^2^) decrease (Figure 3, right pane). Generally, the datasets simulating the instruments with the highest detection thresholds (>100 ppm) can still distinguish between events above background but cannot be used to observe realistic methane mixing ratio values. Simulated instruments with a detection threshold of less than 100 ppm only underestimate mixing ratios by up to 10%, on average (Figure 3, left pane).

#### 3.2.1. Emissions Estimates

**Figure 4:**
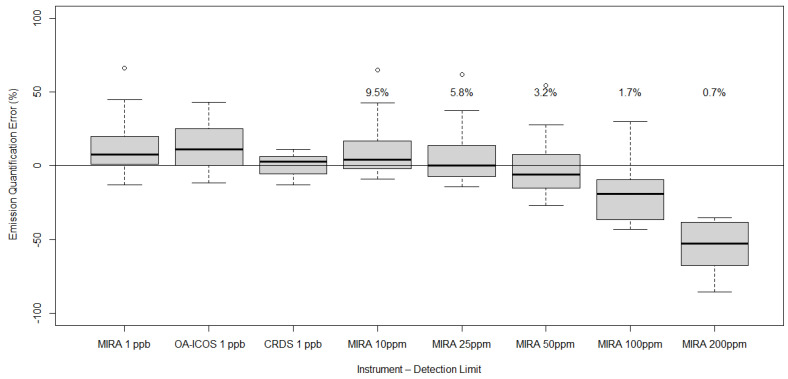
The percentage error in emission quantification for MIRA, OA-ICOS, and CRDS instruments and for the MIRA instrument with simulated detection thresholds of 10, 25, 50, 100, 200, and 300 ppm. For MIRA 10 ppm to MIRA 200 ppm, text indicates the percentage of points that were greater than the threshold value for each of the detection threshold levels.

Methane emissions from 16 emission events were calculated using the 20 min averaged methane mixing ratios from the MIRA, OA-ICOS, and CRDS and meteorological data in Equation (1) (Figure 4). Here, we note the bias in the MIRA and OA-ICOS instruments at +2.94 ppm and +0.23 ppm compared to the CRDS, respectively. Emissions were only calculated when the wind was blowing from the emission point to the instruments (i.e., the measured concentration was above background). Controlled emissions during the emission events were 5.2 kg CH_4_ h^−1^. The average emissions calculated from the CRDS, OA-ICOS, and MIRA data were 5.2 (maximum = 5.8, minimum = 4.5, s.d. = 0.4), 5.8 (maximum = 7.4, minimum = 4.6, s.d. = 0.8), and 5.9 (maximum = 8.6, minimum = 4.5, s.d. = 1.1) kg CH_4_ h^−1^, respectively.

#### 3.2.2. Impact of Higher Detection Threshold on Emission Quantification

Filtering for wind direction, there were 16 20 min emission events used to evaluate the effects of using a higher detection threshold on emission quantification. Using the unfiltered data, the average error of the calculated emission compared to the known emission is +13.3% (95% CI: −7.8%, +51.2%). As the simulated detection threshold increases, the error in the emission quantification becomes more negative (Figure 4). The average error amounts for detection thresholds of 10, 25, 50, 100, and 200 ppm are +11% (95% CI: −7%, +49%), +7% (−13%, +45%), −1% (−23%, +36%), −19% (−42%, +15%), and −56% (−84%, −35%), respectively.

## 4. Discussion

### 4.1. Variability in Instrument Response

Three methane trace-gas analyzers were deployed 20 to 80 m downwind of controlled methane releases that ranged from 0.4 to 5.2 kg methane per hour between 0800 and 1700 on 13 July 2023, at Colorado State University’s METEC facility in Fort Collins. Even though these instruments are designed to do the same thing, i.e., to report dry-mole methane mixing ratios, the instruments’ response at one second resolution was highly variable. The reported average mixing ratios over the nine hours were 10.0 ppm, 10.0 ppm, and 10.1 for the CRDS, OA-ICOS, and MIRA, respectively. However, the maximum observed mixing ratios observed were 83.0 ppm, 301.6 ppm, and 778.9 ppm (Figure 1), with the values reported by the MIRA consistently higher than those by the OA-ICOS, which in turn was higher than those of the CRDS. We are confident that the mixing ratios reported by the MIRA were relatively accurate, i.e., 778.9 ppm reported by the MIRA was at most ±8.4% (713.3 ppm to 844.5 ppm) as it reported methane mixing ratios of 53.7 ppm, 105.6 ppm, and 1084 ppm in response to calibration gases with methane mixing ratios of 50 ppm, 100 ppm, and 1000 ppm, respectively. All calibration standards were accurate to ±5%.

The METEC instruments used in this study vary in age and use and should not be taken as exemplary instruments for each brand and model. The scatter-plots comparing the 20 min averaged data of the three show that the variance between datasets is less than 7% with a bias of less than ±5%. The observations made by the CRDS are the most expected, where mixing ratios downwind of a source emitting 5.2 kg methane per hour would be in the order of 30 ppm in light winds (2 m s^−1^) and an unstable atmosphere (PGSC—B). However, air took 1.5 h to travel through 100 m of ¼” ID PTFE tubing from the inlet to the instrument, which would allow time for mixing and would likely result in a relatively homogenous sample when it arrived at the analyzer. As can be seen from the temporal variability in mixing ratios observed by the CRDS, diffusion was not enough to remove all trends in methane change and acted more as a smoothing mechanism. The CRDS was also housed within a laboratory and effects of any temperature change throughout the day would have been minimized.

Our results show that the average methane concentrations of the CRDS and OA-ICOS are in the best agreement, while average mixing ratios generated using the MIRA have more variability, albeit still within 10%. We suggest that as a smaller instrument, the MIRA is likely more affected by hot/cold air being drawn into the instrument despite the thermally controlled environment. The variability in mixing ratio measurements carries through to the emission quantification where the average emission is biased high by 13% (maximum + 66%, minimum −13%).

The large, short-duration peaks observed by the OA-ICOS and MIRA instruments (Figure 1) suggest that they are detecting a heterogeneous plume generated by the local micrometeorology. We suggest that in turbulent flows, small-scale vortices cause molecules to form small volumes of high-concentration methane instead of discrete molecules travelling individually. Computational fluid dynamic modelling efforts suggest that vortex shedding can result in large changes in gas concentrations over small distances within the gas plume, where aerodynamic obstructions cause pressure changes within the plume and could give rise to a mechanism whereby methane would collect in higher concentrations [26,27,28,29].

The responses of the OA-ICOS and the MIRA were alike, even though the mixing ratios of the OA-ICOS were consistently lower than those of the MIRA (OA-ICOS maxima ≈ 1/10th of the temporally corresponding MIRA maxima). This can be rationalized by considering how the relative pump rates and cavity volumes of the instruments would affect the measurement of small volumes of highly concentrated methane packets in air. The MIRA has a smaller cavity volume (10 cm^3^) and flow rate (0.3 slpm) than the OA-ICOS (100 cm^3^ and 2 slpm). Larger flow rates could result in increased mixing of air, as could the larger volume measurement cavity, resulting in a lower average concentration within the cavity.

As mentioned above, it is likely that the small volumes of high concentrations can only be detected by a methane instrument with a low flow rate through a detection system that is fast enough to respond to rapid fluctuations in methane abundance (hundreds of ppm per second). As a result, we would suggest that optical-cavity-based instruments (cavity size ≤ 10 cm^3^) measuring at ≥2 Hz with air flow rates in the order of ≤0.3 slpm at distances of ≤20 m from the source would be the most likely to register this effect.

### 4.2. Effect of Higher Detection Thresholds

Aside from a better understanding of gas transport in the boundary layer, the observations of this study present a novel perspective on methane detection instrumentation. One aspect of applied science that is essential to reduction of greenhouse gas emission and meeting the climate goals set out by the IPCC and the Global Methane Pledge [1,2,3] is the detection and repair of fugitive methane emissions from oil and gas production and transportation infrastructure. To date, using optical gas imaging cameras is the most effective method of leak detection and repair (LDAR); however, these bi-annual surveys only detect emissions when the LDAR team visits a site. Continuous monitors are thought more likely to be able to alert operators to new fugitive leaks; however, methane sensors are either not fast enough to detect small changes in concentration (metal oxide sensors) or too expensive (optical-cavity-based instruments).

The results of this study suggest that there may be an alternative to deploying trace gas methane instruments that can detect ppm level changes in methane abundance in the atmosphere. Our results suggest that if a small, fast-response, low-flow instrument (optical-cavity-based instruments with cavity size ≤10 cm^3^ measuring at ≥2 Hz with air flow rates in the order of ≤0.3 slpm at distances of ≤20 m from the source) can be placed close enough to a fugitive emission source, it may be able to detect enough high-concentration packets of methane even if it has a higher detection limit than the current ~1 ppm cavity-based instruments. Our analysis suggests that instruments with detection limits of 25 ppm that operate in a way similar to the MIRA could quantify 20 min average concentrations with only a −10% bias and that these averaged concentrations could be used to quantify the emissions to an average of −20% (95% CI: −10, −40%) of a 1 ppm instrument. If the detection limit could be increased, new instruments could be developed using LED-based cavities [30,31], which would reduce instrument cost by factors of ten, resulting in the more widespread deployment of sensors at oil and gas facilities.

Even though this manuscript does not specifically define how a new instrument with a higher detection threshold can be developed, it identifies that if methane can be measured by a fast, small-volume instrument, it is likely that 20 min average concentration values will be representative when compared to an instrument with a lower detection threshold. While it is very likely that the development of an instrument with these capabilities will be difficult, this hypothesis circumvents current assumptions (and associated costs) that sub-ppm observations are essential for high-quality measurements. Moreover, this study envisions a future avenue of investigation for sensor development that has hitherto remained undiscovered and reports that future methane leak detection instrument design and deployment can be optimized using these experimental results, facilitating more accurate and efficient emission quantification.

## 5. Conclusions

This study reports the difference in response of three co-located sub-ppm methane analyzers to changes in methane in the open air. All instruments reported changing methane concentrations at the same time and with average methane mixing ratios of 10 ppm over nine hours; however, the magnitude in response varied by instrument. The CRDS’s 1 Hz methane response was least variable, with maximum values of 83 ppm, while the MIRA’s response at the same time resolution reached 779 ppm. The peak concentrations reported by the MIRA were very short-lived (order of seconds) while the CRDS’s peak concentration lasted for tens of seconds. We suggest this difference at second resolution was caused by instrument configuration, where fast response instruments with low flow rates and located close to the source would be most likely to see short-duration high concentrations of methane in air. Even though we cannot explicitly explain the cause of the high concentrations, these could result from either the effects of micrometeorology or a feature of the methane release system. Regardless of the cause, this implies that an instrument operating in a similar way to the MIRA (an optical-cavity-based instrument with cavity size ≤10 cm^3^ measuring at ≥2 Hz with air flow rates in the order of ≤0.3 slpm at distances of ≤20 m from the source) could detect enough of the high-concentration events to generate a representative average methane mixing ratio. This has implications for using instrumentation with higher detection thresholds, resulting in the reduction in cost to measure methane emissions and providing a mechanism for the widespread deployment of effective leak detection and repair programs at all oil and gas infrastructure. Further work should investigate the enhancements to the experimental design and include expanding the sample size (duration of measurement, size of emission, distance from the emission point), and explore alternative types of methane detection instruments (metal oxide, LED-based, tunable diode laser spectrometers). Other additional work could include investigating the interplay of sample volume, flow rate, and sampling rate since these parameters appear to be underlying drivers when measuring high-frequency concentration fluctuations that have been observed by this study.

## Figures and Tables

**Table 1:** Time, location, and size of controlled methane releases at the METEC site on 13 July 2023.

Date	Release Start	Release End	Location: Pad	Emission Rate (kg CH_4_ h^−1^)
10 July 2023	9:56	10:55	5	5.3
12 July 2023	10:50	12:10	5	5.2
12 July 2023	12:13	13:12	5	5.2
13 July 2023	8:14	9:31	1	0.4
13 July 2023	9:37	10:49	5	5.2
13 July 2023	10:51	12:00	1	0.4
13 July 2023	12:13	13:12	5	5.2
13 July 2023	13:28	14:22	5	5.2
13 July 2023	14:52	15:47	5	5.2

**Table 2:** The slope, intercept, *R*^2^, and bias calculated using the scatter-plots of the 20 min reported averages from the CRDS, OA-ICOS, and MIRA instruments (Figure 2).

		CRDS	OA-ICOS	MIRA			CRDS	OA-ICOS	MIRA
		Intercept (ppmv)					*R* ^2^		
CRDS	Slope	-	−0.09	−0.36	CRDS	Bias (%)	-	0.97	0.95
OA-ICOS		1.01	-	0.44	OA-ICOS		0.23	-	0.93
MIRS		1.05	0.95	-	MIRA		2.94	−3.07	-

## Data Availability

Data can be accessed at Colorado State University’s Mountain Scholar data repository.

## References

[1] Intergovernmental Panel on Climate Change (2014). IPCC Climate Change 2013—The Physical Science Basis: Working Group I Contribution to the Fifth Assessment Report of the Intergovernmental Panel on Climate Change.

[2] Pörtner H.-O., Roberts D.C., Tignor M., Poloczanska E.S., Mintenbeck K., Alegría A., Craig M., Langsdorf S., Löschke S., Möller V. (2022). IPCC Climate Change 2022: Impacts, Adaptation and Vulnerability. Contribution of Working Group II to the Sixth Assessment Report of the Intergovernmental Panel on Climate Change.

[3] Nisbet E.G., Fisher R.E., Lowry D., France J.L., Allen G., Bakkaloglu S., Broderick T.J., Cain M., Coleman M., Fernandez J. (2020). Methane Mitigation: Methods to Reduce Emissions, on the Path to the Paris Agreement. Rev. Geophys..

[4] Seinfeld J.H., Pandis S.N. (2016). Atmospheric Chemistry and Physics: From Air Pollution to Climate Change.

[5] Denmead O.T. (2008). Approaches to Measuring Fluxes of Methane and Nitrous Oxide between Landscapes and the Atmosphere. Plant Soil.

[6] Pihlatie M.K., Christiansen J.R., Aaltonen H., Korhonen J.F.J., Nordbo A., Rasilo T., Benanti G., Giebels M., Helmy M., Sheehy J. (2013). Comparison of Static Chambers to Measure CH4 Emissions from Soils. Agric. For. Meteorol..

[7] Collier S.M., Ruark M.D., Oates L.G., Jokela W.E., Dell C.J. (2014). Measurement of Greenhouse Gas Flux from Agricultural Soils Using Static Chambers. J. Vis. Exp..

[8] Aneja V.P., Blunden J., Claiborn C.S., Rogers H.H., Barnes I., Rudzinski K.J. (2006). Dynamic Chamber System to Measure Gaseous Compounds Emissions and Atmospheric-Biospheric Interactions. Environmental Simulation Chambers: Application to Atmospheric Chemical Processes.

[9] Caulton D.R., Li Q., Bou-Zeid E., Fitts J.P., Golston L.M., Pan D., Lu J., Lane H.M., Buchholz B., Guo X. (2018). Quantifying Uncertainties from Mobile-Laboratory-Derived Emissions of Well Pads Using Inverse Gaussian Methods. Atmos. Chem. Phys..

[10] Edie R., Robertson A.M., Field R.A., Soltis J., Snare D.A., Zimmerle D., Bell C.S., Vaughn T.L., Murphy S.M. (2020). Constraining the Accuracy of Flux Estimates Using OTM 33A. Atmos. Meas. Tech..

[11] Robertson A.M., Edie R., Field R.A., Lyon D., McVay R., Omara M., Zavala-Araiza D., Murphy S.M. (2020). New Mexico Permian Basin Measured Well Pad Methane Emissions Are a Factor of 5–9 Times Higher Than U.S. EPA Estimates. Environ. Sci. Technol..

[12] Riddick S.N., Mauzerall D.L., Celia M., Allen G., Pitt J., Kang M., Riddick J.C. (2020). The Calibration and Deployment of a Low-Cost Methane Sensor. Atmos. Environ..

[13] Zhu L.-Y., Ou L.-X., Mao L.-W., Wu X.-Y., Liu Y.-P., Lu H.-L. (2023). Advances in Noble Metal-Decorated Metal Oxide Nanomaterials for Chemiresistive Gas Sensors: Overview. Nano-Micro Lett..

[14] Ou L.-X., Liu M.-Y., Zhu L.-Y., Zhang D.W., Lu H.-L. (2022). Recent Progress on Flexible Room-Temperature Gas Sensors Based on Metal Oxide Semiconductor. Nano-Micro Lett..

[15] Riddick S.N., Ancona R., Cheptonui F., Bell C.S., Duggan A., Bennett K.E., Zimmerle D.J. (2022). A Cautionary Report of Calculating Methane Emissions Using Low-Cost Fence-Line Sensors. Elem. Sci. Anthr..

[16] Golston L., Aubut N., Frish M., Yang S., Talbot R., Gretencord C., McSpiritt J., Zondlo M. (2018). Natural Gas Fugitive Leak Detection Using an Unmanned Aerial Vehicle: Localization and Quantification of Emission Rate. Atmosphere.

[17] Gålfalk M., Nilsson Påledal S., Bastviken D. (2021). Sensitive Drone Mapping of Methane Emissions without the Need for Supplementary Ground-Based Measurements. ACS Earth Space Chem..

[18] Commane R., Hallward-Driemeier A., Murray L.T. (2023). Intercomparison of Commercial Analyzers for Atmospheric Ethane and Methane Observations. Atmos. Meas. Tech..

[19] Peltola O., Mammarella I., Haapanala S., Burba G., Vesala T. (2013). Field Intercomparison of Four Methane Gas Analyzers Suitable for Eddy Covariance Flux Measurements. Biogeosciences.

[20] O’Keefe A. (1998). Integrated Cavity Output Analysis of Ultra-Weak Absorption. Chem. Phys. Lett..

[21] Engeln R., Berden G., Peeters R., Meijer G. (1998). Cavity Enhanced Absorption and Cavity Enhanced Magnetic Rotation Spectroscopy. Rev. Sci. Instrum..

[22] Von Bobrutzki K., Braban C.F., Famulari D., Jones S.K., Blackall T., Smith T.E.L., Blom M., Coe H., Gallagher M., Ghalaieny M. (2010). Field Inter-Comparison of Eleven Atmospheric Ammonia Measurement Techniques. Atmos. Meas. Tech..

[23] Laubach J., Kelliher F.M., Knight T.W., Clark H., Molano G., Cavanagh A. (2008). Methane Emissions from Beef Cattle—A Comparison of Paddock- and Animal-Scale Measurements. Aust. J. Exp. Agric..

[24] Flesch T.K., Harper L.A., Powell J.M., Wilson J.D. (2009). Inverse-Dispersion Calculation of Ammonia Emissions from Wisconsin Dairy Farms. Trans. ASABE.

[25] US EPA (1995). Industrial Source Complex (ISC3) Dispersion Model.

[26] Sauer J.A., Muñoz-Esparza D., Canfield J.M., Costigan K.R., Linn R.R., Kim Y.-J. (2016). A Large-Eddy Simulation Study of Atmospheric Boundary Layer Influence on Stratified Flows over Terrain. J. Atmos. Sci..

[27] Muñoz-Esparza D., Sauer J.A., Linn R.R., Kosović B. (2016). Limitations of One-Dimensional Mesoscale PBL Parameterizations in Reproducing Mountain-Wave Flows. J. Atmos. Sci..

[28] Smith C.M., Skyllingstad E.D. (2009). Investigation of Upstream Boundary Layer Influence on Mountain Wave Breaking and Lee Wave Rotors Using a Large-Eddy Simulation. J. Atmos. Sci..

[29] Tao R., Ren H., Wang D., Bai X. (2022). Research on Smoke Simulation with Vortex Shedding. PLoS ONE.

[30] Wastine B., Hummelgård C., Bryzgalov M., Rödjegård H., Martin H., Schröder S. (2022). Compact Non-Dispersive Infrared Multi-Gas Sensing Platform for Large Scale Deployment with Sub-Ppm Resolution. Atmosphere.

[31] Li N., Tao L., Yi H., Kim C.S., Kim M., Canedy C.L., Merritt C.D., Bewley W.W., Vurgaftman I., Meyer J.R. (2021). Methane Detection Using an Interband-Cascade LED Coupled to a Hollow-Core Fiber. Opt. Express.

